# Physiological and Proteomic Analysis of Different Molecular Mechanisms of Sugar Beet Response to Acidic and Alkaline pH Environment

**DOI:** 10.3389/fpls.2021.682799

**Published:** 2021-06-09

**Authors:** Gui Geng, Gang Wang, Piergiorgio Stevanato, Chunhua Lv, Qiuhong Wang, Lihua Yu, Yuguang Wang

**Affiliations:** ^1^National Sugar Crop Improvement Centre, College of Advanced Agriculture and Ecological Environment, Heilongjiang University, Harbin, China; ^2^Heilongjiang Sugar Beet Center of Technology Innovation, College of Advanced Agriculture and Ecological Environment, Heilongjiang University, Harbin, China; ^3^College of Life Sciences, Heilongjiang University, Harbin, China; ^4^DAFNAE, Dipartimento di Agronomia, Animali, Alimenti, Risorse Naturali e Ambiente, Università degli Studi di Padova, Padova, Italy

**Keywords:** sugar beet, soil pH, acid stress, TMT, proteomics

## Abstract

Soil pH is a major constraint to crop plant growth and production. Limited data are available on sugar beet growth status under different pH conditions. In this study, we analyzed the growth status and phenotype of sugar beet under pH 5, pH 7.5, and pH 9.5. It was found that the growth of sugar beet was best at pH 9.5 and worst at pH 5. The activities of superoxide dismutase (SOD) and peroxidase (POD) in leaves and roots increased as pH decreased from 9.5 to 5. Moreover, compared with pH 9.5, the levels of soluble sugar and proline in leaves increased significantly at pH 5. To explore the mechanisms of sugar beet response to different soil pH environments, we hypothesized that proteins play an important role in plant response to acidic and alkaline pH environment. Thus, the proteome changes in sugar beet modulated by pH treatment were accessed by TMT-based quantitative proteomic analysis. A total of three groups of differentially expressed proteins (DEPs) (pH 5 vs. pH 7.5, pH 9.5 vs. pH7.5 and pH 5 vs. pH 9.5) were identified in the leaves and roots of sugar beet. Several key proteins related to the difference of sugar beet response to acid (pH 5) and alkaline (pH 9.5) and involved in response to acid stress were detected and discussed. Moreover, based on proteomics results, QRT-PCR analysis confirmed that expression levels of three N transporters (*NTR1*, *NRT2.1*, and *NRT2.5*) in roots were relatively high under alkaline conditions (pH 9.5) compared with pH 5 or pH 7.5. The total nitrogen content of pH 9.5 in sugar beet was significantly higher than that of pH 7.5 and pH 5. These studies increase our understanding of the molecular mechanism of sugar beet response to different pH environments.

## Introduction

Soil pH is commonly considered a dominant factor affecting plant growth and development, and unfavorable soil pH decreases the quantity and quality of crop yield (Tang et al., 2001). Subsurface soil acidity impairs root growth of sensitive crops and hence may reduce plant access to water reserves in the subsurface soil layer (Tang et al., 2001). Furthermore, aluminum (Al) toxicity constraints root elongation in acid soils (Haling et al., 2011). Also, high soil pH imbalances macro-, and microelements in soil that lead to physiological depression of plants. For example, iron and zinc availability for plants is influenced by high pH (Sisó-Terraza et al., 2016; Dmitriev et al., 2019). Moreover, soil pH is widely considered as a universal indicator of the structural features of bacterial communities and is closely associated with populations of soil microbial communities (Zheng et al., 2019). Usually, the optimal pH level for plant growth varies greatly in different plant species. Therefore, understanding the molecular mechanisms of plant response to unfavorable pH conditions and identification of resistance gene candidates will help in the breeding of improved cultivars.

When plants are exposed to different pH conditions, they exhibit different physiological and molecular responses. Some studies investigated the plant antioxidant responses of roots and leaves to low pH (Zhang et al., 2015; Long et al., 2019) and found that pH 2.5-induced accumulation of H_2_O_2_ and malonaldehyde (MDA) in rice roots was accompanied by decreased antioxidant enzyme activities (Zhang et al., 2015). Low pH also affected methylglyoxal (MG) metabolisms, which played a role in low pH-tolerance in higher plants through the detoxification of MG by glyoxalase (Gly) I and II (Long et al., 2019). Recently, it was reported that iron walnut growth was better in pH 4-5 and pH 5-6 treatments than in pH 3-4 and pH 6-7 treatments. Transcriptome analyses revealed that the pathways involved in polyamine metabolisms participated in iron walnut acid stress tolerance (Luo and Liu, 2019). Moreover, under alkaline conditions, high rhizosphere pH inhibits plant growth by imposing an adverse effect on roots elongation and disrupting cellular ionic homeostasis and pH. Several recent reports show that plasma membrane H^+^-ATP synthase (H^+^-ATPase) plays an important role in the adaptation of plants to alkaline stress by acidifying the rhizosphere *via* plasma membrane through mediating proton secretion. Several factors are found to regulate the activities of H^+^-ATPase (Fuglsang et al., 2007; Yang et al., 2010). For example, the protein kinase PROTEIN KINASE SOS2-LIKE5 (PKS5) regulates proton secretion by preventing the interaction between 14-3-3 proteins and the plasma membrane H^+^-ATPase (Fuglsang et al., 2007). However, other adaptive mechanisms by plants in response to unfavorable pH have not yet been fully explored.

*Beta vulgaris* (sugar beet) is one of the most important industrial crops for sugar or bioethanol production and is considered one of the most salt-tolerant crops (Lv et al., 2019). Several studies on the response to salt stress have been conducted at physiological and molecular levels using proteome and transcriptome techniques (Geng et al., 2019; Wang et al., 2019). However, studies have never been conducted on sugar beet response to different pH. In this study, sugar beet shown different phenotypic and physiological changes under different pH conditions. We hypothesized that sugar beet had different molecular mechanisms to respond to different pH environments, and several proteins played a key role in different response of sugar beet. A comparative physiology and proteomic analysis of sugar beet under different pH conditions was conducted, and key proteins and pathways important to sugar beet adaptation and tolerance to unfavorable pH were identified. The results provide a valuable genetic resource for further investigation of the molecular mechanisms underlying unfavorable pH tolerance in sugar beet that may be transferred to other crops.

## Materials and Methods

### Plant Material and Growth Condition

Seeds of sugar beet (*Beta vulgaris* cv. H004 from Advanta Company of Netherlands) were selected as plant materials. It is a moderate variety in response to soil pH and widely used in Heilongjiang Province of China. The pH of the original soil (vermiculite: washed sands: black soil, 1:1:3) used for planting was measured and adjusted to generate a series of pH soils (pH 5.0, pH 7.5, and pH 9.5) by adding the appropriate amount of H_2_SO_4_ or Na_2_CO_3_ (Table 1). To avoid a difference in salt content in the three treatments, different levels of NaCl and Na_2_SO_4_ were added to ensure 50 mM Na^+^ for each treatment (Table 1). Twenty seeds were sown in a pot containing 650 g soil with different pH. In order to ensure the normal growth of plants, only four seedlings with the same growth status were retained in each pot after 5 days of sowing. Each pot was considered as a single replicate, and there were three biological replicates for each pH treatment. Sugar beet seedlings were grown in a greenhouse under a 14-h light (24°C)/10-h dark (19°C) photoperiod with 75% relative humidity and a photosynthetic photon flux density of 450 μmol m^–2^ s^–1^. Each pot was irrigated with 50 mL Hoagland nutrient solution every 10 days. After 25 days of growth, the leaves and roots of seedlings were harvested, and the roots of seedlings were washed with different pH water (pH 5.0, pH 7.5, and pH 9.5). Then, these samples were immediately frozen in liquid nitrogen and stored at −80°C for subsequent physiological index and proteome analyses. Each treatment consisted of three biological replicates, and four plants were combined for each replicate.

**TABLE 1:** Salt content and soil electrical conductivity (EC) of different pH treatment.

Treatment	Salinity (Na^+^ mM/kg soil)	NaCl (g/kg soil)	Na_2_SO_4_ (g/kg soil)	Na_2_CO_3_ (g/kg soil)	H_2_SO_4_ (g/kg soil)	EC (ds m^–1^)
pH 5.0	50	1.46	1.78	–	1.84	2.74 ± 0.39
pH 7.5	50	1.46	1.78	–	–	2.37 ± 0.07
pH 9.5	50	–	–	2.65	–	2.13 ± 0.05

### Growth Status and Physiological Index Analysis

For determination of fresh weight, sugar beet seedlings were weighed after being washed with sterile distilled water. The leaf area was obtained by an LI-3000C portable area meter (LI-COR Biosciences, Lincoln, NE, United States). After harvesting, the root area of seedlings was estimated by an optical scanner-based image analysis system (WinRHIZO). The photosynthesis parameters such as photosynthetic rate (Pn), stomatal conductance, and CO_2_ concentration inside were measured on the first fully expanded leaf of seedlings using an LI-6400 portable photosynthesis system (LI-COR Biosciences, Lincoln, NE, United States) on harvest day (in the morning, 9:00 am). Chlorophyll concentrations in leaves were assessed using the method given by Kaur et al. (2016). Leaf samples (0.5 g) were homogenized with 10 mL of acetone (80% v/v) followed by centrifuging at 15,000 *g* for 3 min. The supernatant was then collected, and absorbance was measured at 663 and 645 nm. The SOD and POD activities were assayed using the method published earlier by the laboratory (Wang et al., 2019). Malondialdehyde (MDA) content was determined by the thiobarbituric acid (TBA) reaction using the method described by Chołuj et al. (2014). Soluble sugar and proline content were determined using the method given in Geng et al. (2020). The total nitrogen content of sugar beet seedlings was measured using the method described by Wang et al. (2017a).

### Protein Extraction

Leaf or root samples (2 g) were ground by liquid nitrogen, and the powder was transferred to a 10 mL centrifuge tube. The extraction buffer (100 mM EDTA, 100 mM Tris, 50 mM Borax, 50 mM Vitamin C, 1% PVPP (W/V), 1% Triton-100 (V/V), 2% 2-mercaptoethanol (V/V), 30% sucrose (W/V), pH 8.0) was then added to the powder and vortexed at 4°C for 10 min. An equal amount of pre-cooled tris-saturated phenol (pH 8.0) was added and the mixture was further vortexed at 4°C for 10 min. After the mixture was centrifuged for 20 min at 4°C and 12,000 *g*, the upper stage of phenol was transferred to a new centrifuge tube. An equal amount of extraction buffer was then added to the centrifuge tube and the mixture was vortexed at 4°C for 10 min. After centrifugation at 4°C for 20 min with 12,000 *g*, the upper stage of phenol was collected. Proteins were precipitated by adding five-volume of 0.1 M ammonium acetate- saturated methanol at −20°C for 12 h. After centrifugation, the protein precipitate was washed twice with cold 90% acetone and redissolved in dissolution buffer with 8 M urea, 1% SDS, and cocktail. Subsequently, the redissolved protein sample was sonicated, and the supernatant was extracted after centrifugation at 14,000 *g* for 20 min (Liu et al., 2018). Protein quantification in the solution was performed using BCA protein quantitative kit (Thermo Fisher Scientific, United States).

### Protein Digestion, TMT Labeling, and HPLC Fractionation

A total of 100 μg of protein from each sample was taken for protein digestion, and the protein samples were dissolved in 100 mM triethylammonium bicarbonate (TEAB) to a total volume of 100 μL. The samples were then treated with 10 mM tris (2-carboxyethyl) phosphine (TCEP) for 1 h at 37°C. Afterward, they were alkylated with 40 mM iodoacetate at room temperature in darkness for 40 min. After adding 600 μL of pre-cooled acetone, the samples were centrifuged at 10,000 *g* for 20 min, and the precipitate was dissolved in 100 μL 50mM TEAB solution. Subsequently, the samples were digested with trypsin (Promega, United States) at an enzyme/substrate ratio of 1:50 at 37°C for 14 h (Guo et al., 2019). The digested samples were quantified by Thermo Scientific Pierce Quantitative Fluorometric Peptide Assay. Labeling of the peptides with TMT 10 plex tags Kit (Thermo Fisher Scientific) was performed as described in the manufacturers’ manual. An equal amount of each sample (100 μg) was taken for tandem mass tag labeling. The replicate samples of pH 5.0 roots were labeled with TMT-126, TMT-127N, and TMT-127C, and reagents TMT-128N, TMT-128C, and TMT-129N for roots at pH 7.5. Labeling with TMT-130N, TMT-13,0C, and TMT-131 was applied for roots at pH 9.5. Also, in the process of leaf sample labeling, the replicate samples of pH 5.0 leaves were labeled with TMT-126, TMT-127N, and TMT-127C. The reagents TMT-128N, TMT-128C, and TMT-129N were for leaves at pH 7.5, and TMT-130N, TMT-130C, and TMT-131 were applied for leaves at pH 9.5. An equal amount of each labeled leaves (or roots) sample was mixed desalted and dried by vacuum centrifugation at 1,000 g for 30 min. The peptides mixtures were fractionated using high-pH reverse-phase HPLC with an ACQUITY UPLC BEH C18 Column (1.7 μm, 2.1 mm × 150 mm) (Waters, United States). Briefly, buffer A and buffer B consisted of 2% acetonitrile and 80% acetonitrile, respectively, and both buffers were adjusted to pH 10 with ammonium hydroxide. A total of 30 fractions were collected from each sample and the peptides were then combined into 15 fractions and dried by vacuum centrifugation.

### LC-MS/MS and Data Analysis

Each fraction was resuspended with 30 μL of solvent A (2% acetonitrile, 0.1% formic acid), and LC-MS/MS analysis was performed using a Thermo EASY-1200 UPLC system coupled to a Q Exactive HF-X Hybrid Quadrupole Orbitrap (Thermo Fisher Scientific, United States). The peptides were eluted using a four phases linear gradient of solvent B (0.1% formic acid in 98% ACN) and the following gradient parameters were used: about 5% B over 1-40 min; about 23% B over 41-50 min; about 29% B over 51-56 min; about 38% B over 57-58 min; about 48% B over 58-59 min; about 100% B over 60-65 min; then held at 100% for 3 min. The parameters were as follows: parent ion scanning range recorded in the 350-1300 m/z range. The mass-to-charge ratio of the fragments of peptides and polypeptides was collected as follows: 20 fragment maps [MS2 scan, high energy collision dissociation (HCD)] were acquired after each full scan, which employed primary MS resolution of 60,000, automatic gain control (AGC) target values of 3e6, Level 1 maximum injection time (MIT) of 20 ms, and secondary MS resolution of 30,000, a target AGC value of 1e5, and Level 2 MIT of 50 ms (MS2 Activation Type: HCD; Isolation window: 1.6 e5; Normalized collision energy: 35) (Guo et al., 2019). The MS/MS spectra output was obtained as a raw file and searched using the Proteome Discoverer software 2.2 (Proteome Discoverer Version 2.2, Thermo Fisher Scientific Inc., 2012) against the GCF_000511025.2_RefBeet-1.2.2_protein database (*Beta Vulgaris* L.). Each search was specified to include trypsin digestion (allowing up to two missed cleavages). The oxidation of methionine and N-terminal acetylation were set as a dynamic modification and cysteine alkylation as iodoacetamide. Mass tolerance was 20 ppm for precursor ions and 0.02 Da for fragment ions. At least two unique peptides were used for protein quantification, and the method of normalization on protein median was applied to correct experimental bias. Differentially expressed proteins (DEPs) were screened from the total identified proteins, based on the following criteria: *P*-values Students *t*-test smaller than 0.05 and a fold-change of >1.2 or <0.83.

### Bioinformatics Analysis

The differentially expressed proteins (DEPs) were categorized with Gene Ontology (GO) database, UniProt database, and KEGG (Kyoto Encyclopedia of Genes and Genomes) database. Principal components analysis (PCA) and Heatmap were performed using SIMCA-P (version 11.5) and Genesis software, respectively. To further explore the functions of differentially expressed proteins, enrichment of GO analysis was performed using Goatools software^1^ by Fisher’s exact test (Lu et al., 2012). Only functional categories with *P*-values < 0.05 were considered to have significant enrichment.

### Quantitative Real-Time PCR Validation

Total RNA from sugar beet tissues was extracted using a TRIzol reagent according to the manufacturer’s instruction (Life Technology, United States). First-strand cDNAs were synthesized using a reverse transcription kit from Toyobo Company (Japan). QRT-PCR was performed using the SYBR Premix Ex Taq Kit (TaKaRa, China) and a Bio-Rad Quantitative PCR system (Bio-Rad, United States). *18S rRNA* was used as an internal control to normalize all data, and the reactions were run as follows: 20 s at 95°C, followed by 40 cycles of 95°C for 15 s, 55°C for 30 s and then 72°C for 20 s in 96-well optical reaction plates. All reactions contained three biological replicates, and the primers are listed in Supplementary Table 1. The semi-quantitative RT-PCR process was based on the previous papers published in our laboratory (Wang et al., 2012), and the primers are listed in Supplementary Table 1.

### Statistical Analysis

The data obtained were expressed as means and standard errors, and all of the experiments were repeated three times. Analysis of variance (ANOVA) between the physiological parameters of different pH treatment were performed using SPSS 13.0 (SPSS Inc., Chicago, IL, United States). All the histograms were made by graphpad prism (8.0) software. The qRT-PCR data were subjected to ANOVA analyses.

## Results

### Effect of Different Soil pH on Sugar Beet Growth Status

**FIGURE 1:**
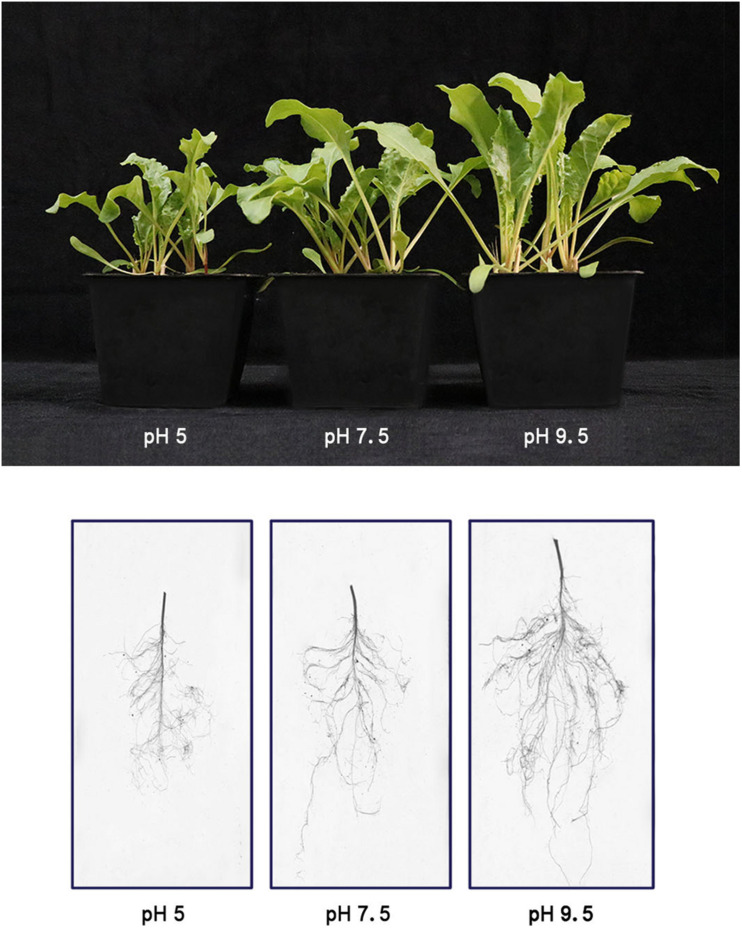
The phenotype and growth status of sugar beet in three pH soils.

**FIGURE 2:**
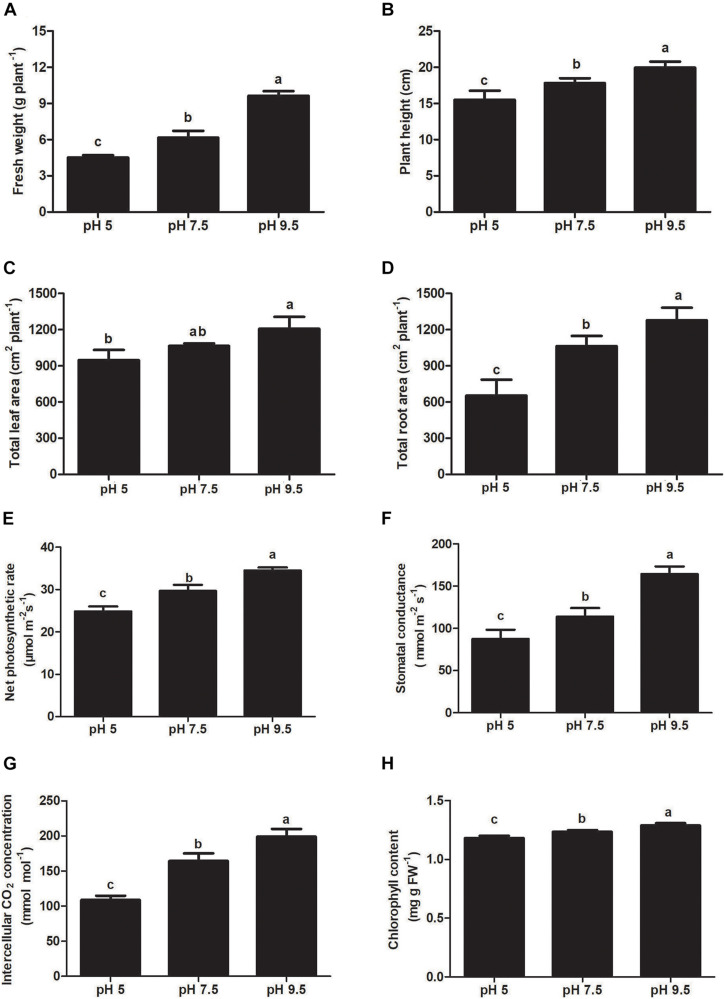
Effects of soil pH on fresh weight **(A)**, plant height **(B)**, leaf area **(C)**, and total root length **(D)**, net photosynthetic rate **(E)**, intercellular CO2 concentration **(F)**, stomatal conductance **(G)**, and chlorophyll content **(H)** in sugar beet. Values represent the means of three biological replicates. Different letters indicate significantly different at *p* < 0.05.

Sugar beet seeds were sown in pH 5, pH 7.5, and pH 9.5 soil and grown for 25 days, seedlings showed different phenotypes and growth state (Figure 1). Under the high pH condition of 9.5, sugar beet seedlings exhibited the best growth phenotype (Figure 1). Instead, the growth of aerial part and roots was significantly inhibited under the acidic condition of pH 5 compared with pH 7.5 and pH 9.5 (Figure 1). The moderate growth state of seedlings was found to be under neutral pH 7.5. Some growth indexes of sugar beet (fresh weight, plant height, leaf area, and root area) were the highest at pH 9.5 and the lowest at pH 5 (Figures 2A-D). For example, compared with seedlings at pH 7.5 and pH 9.5, the fresh weight of plants under pH 5.5 decreased by 12.90% and 22.19%, respectively (Figure 2A). These results indicated that sugar beet is an alkali loving crop adapted to high pH, and the acid condition would seriously inhibit its growth.

### Effect of Different Soil pH on Sugar Beet Photosynthesis, Antioxidant System, and Osmotic Adjustment Substance

To investigate the effects of each pH treatment on plant photosynthesis, the related indicators were analyzed. Chlorophyll content, net photosynthetic rate, intercellular CO_2_ concentration, and stomatal conductance decreased significantly as pH decreased from 9.5 to 5 (Figures 2E-H). These photosynthetic indicators in leaves were the highest at pH 9.5 as compared with pH 5 and pH 7.5. The photosynthetic rate at pH 9.5 was 16.33% and 38.81% higher than that at pH 7.5 and pH 5, respectively. Our results showed that low soil pH can exert strongly negative effects on photosynthesis and a high pH promotes it in sugar beet seedlings.

Malonaldehyde content is usually one of the important indicators of stress-caused plasma membrane oxidative injury. In this study, MDA levels increased as pH decreased from 9.5 to 5 in leaves and roots (Table 2). These results showed that membrane damage increased with the decrease in pH value. Furthermore, the activities of antioxidant enzymes implicated in the detoxification of reactive oxygen species (ROS) are thought to be indicators for plant response to several stresses. The activities of SOD and POD in leaves and roots increased as pH decreased from 9.5 to 5 (Table 2). Membrane oxidative damage caused by low pH will lead to plant activation of the antioxidant enzyme system to cope with stress. Moreover, compared with pH 9.5, the levels of soluble sugar and proline in the sugar beet leaves increased significantly in pH 5 (Table 2). Thus, sugar beet tends to synthesize higher levels of soluble organic solutes to cope with acid stress.

**TABLE 2:** The activities of SOD and POD, and the amounts of MDA, soluble sugar and proline occurring in leaves and roots of sugar beet treated with different soil pH.

Treatment	SOD (U⋅mg^–1^ protein)		POD (μ mol⋅min^–1^ mg^–1^ protein)		MDA (μ mol⋅g^–1^ FW)		Soluble sugar (g⋅g^–1^ FW)		Proline (g⋅g^–1^ FW)	
	Leaf	Root	Leaf	Root	Leaf	Root	Leaf	Root	Leaf	Root
pH 5.0	89.84±1.40^a^	15.24±0.22^a^	0.45±0.0106^a^	0.49±0.0313^a^	0.51±0.01^a^	0.92±0.1556^a^	4.99±0.20^a^	28.29±2.15^a^	38.57±3.07^a^	17.30±1.44^a^
pH 7.5	81.82±1.34^b^	12.98±0.15^b^	0.33±0.0127^b^	0.35±0.0088^b^	0.46±0.01^b^	0.63±0.0155^b^	3.61±0.42^b^	16.99±0.54^b^	21.43±0.12^b^	17.77±0.71^a^
pH 9.5	78.41±2.09^c^	10.04±0.55^c^	0.17±0.0066^c^	0.29±0.0202^b^	0.36±0.04^c^	0.49±0.0023^b^	3.15±0.25^b^	15.19±0.62^b^	15.57±0.95^c^	17.10±0.72^a^

*Different letters indicate significantly different at p < 0.05.*

### Proteomic Analysis

To uncover the mechanisms of sugar beet response to different soil pH environments, proteome changes modulated by pH treatment were accessed by TMT-based quantitative proteomic analysis. The proteins were extracted and digested with trypsin followed by TMT labeling. After quality validation, a total of 47807 and 53901 identified peptides were detected in leaves and roots, respectively (Supplementary Tables 2, 3). Approximately 7044 proteins were identified in roots and 7720 in leaves (Supplementary Table 4). To analyze the contribution of differentially expressed proteins to proteomics variance and evaluate the reliability of harvested samples, PCA on all samples from three replicates of three pH treatments was performed. As indicated in Supplementary Figure 1, three pH treatments were obviously separated and three replicates of each treatments were flocked together, indicating sample collection was reliable. To identify sugar beet protein expression profiles in response to different pH conditions, a total of three groups of differentially expressed proteins (DEPs) (pH 5 vs. pH 7.5, pH 9.5 vs. pH 7.5, and pH 5 vs. pH 9.5) were screened in leaves and roots (Supplementary Tables 5, 6). The DEP_*S*_ groups of pH 5 vs. pH 7.5 and pH 9.5 vs. pH7.5 were used to explore the different sugar beet response to acid (pH 5) and alkaline (pH 9.5) conditions. Furthermore, it can be seen from the previous results that sugar beet had the best growth state at pH 9.5 condition and pH 5 seriously inhibited plant growth. Therefore, to fully understand the effect of acid on sugar beet growth and its response mechanism to acid stress, the DEP_*S*_ identified in pH 5 vs. the pH 9.5 group were also detected.

### Identification of Differentially Expressed Proteins

**FIGURE 3:**
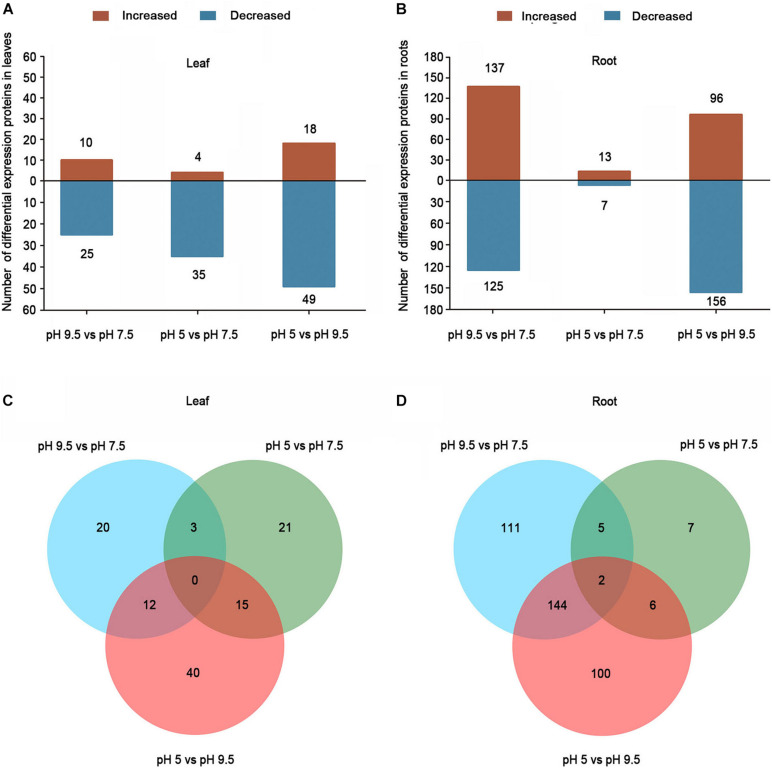
Differentially expressed proteins (DEPs) in the leaves and roots of sugar beet in different pH comparison groups. Numbers of DEPs in leaf **(A)** and root **(B)** at different salt treatments. Venn diagrams of DEGs among different pH comparison groups in leaf **(C)** and root **(D)**.

Differentially expressed proteins were defined as those with a 1.2-fold or 0.83-fold change in relative abundance (*p* < 0.05) between pH 5 and pH 7.5, pH 9.5 and pH7.5, and pH 5 and pH 9.5 (Supplementary Tables 5-8). Furthermore, we conducted a hierarchical cluster analysis based on the abundance of differentially expressed proteins in each group (Supplementary Figure 2), and the results supported that the DEPs screened through our experiment were accurate (Supplementary Figure 2). In total, 35 (10 up-regulated, 25 down-regulated), 39 (4 up-regulated, 35 down-regulated), and 67 (18 up-regulated, 49 down-regulated) DEPs were identified in leaves comparing pH 9.5 vs. pH 7.5, pH 5 vs. pH 7.5, and pH 5 vs. pH 9.5, respectively (Figure 3A and Supplementary Tables 5, 7). In total, 262 (137 up-regulated, 125 down-regulated), 20 (13 up-regulated, 7 down-regulated), and 252 (96 up-regulated, 156 down-regulated) DEPs were found in the groups of pH 9.5 vs. pH 7.5, pH 5 vs. pH 7.5, and pH 5 vs. pH 9.5 in roots, respectively (Figure 3B and Supplementary Tables 6, 8). As roots are the pivotal tissue used to sense and respond to pH change in soils, the number of DEPs identified in roots is more than in leaves. Furthermore, the largest number of DEPs was identified in the comparison of pH 5 vs. pH 9.5 in the leaves or roots of sugar beet. This result is consistent with the phenotypic data of plants under different pH conditions, the largest phenotypic difference was detected between pH 5 and pH 9.5. To identify the common specifically changed proteins in the comparison of the three groups, a Venn diagram was generated (Figures 3C,D). There were no DEPs common to all three groups in leaves. Only two DEPs common to all three groups were found in roots. Furthermore, the overlapping analysis found that there were 20, 21, and 40 unique DEPs in the leaves of pH 9.5 vs. pH 7.5, pH 5 vs. pH 7.5, and pH 5 vs. pH 9.5, respectively, and a total of 111, 7, 150 DEPs were unique to each pH treatment in roots (Figures 3C,D). These DEPs may have contributed to the phenotypic discrepancy between different pH conditions.

### Functional Classification and Gene Ontology (GO) Enrichment of Differentially Expressed Proteins

**FIGURE 4:**
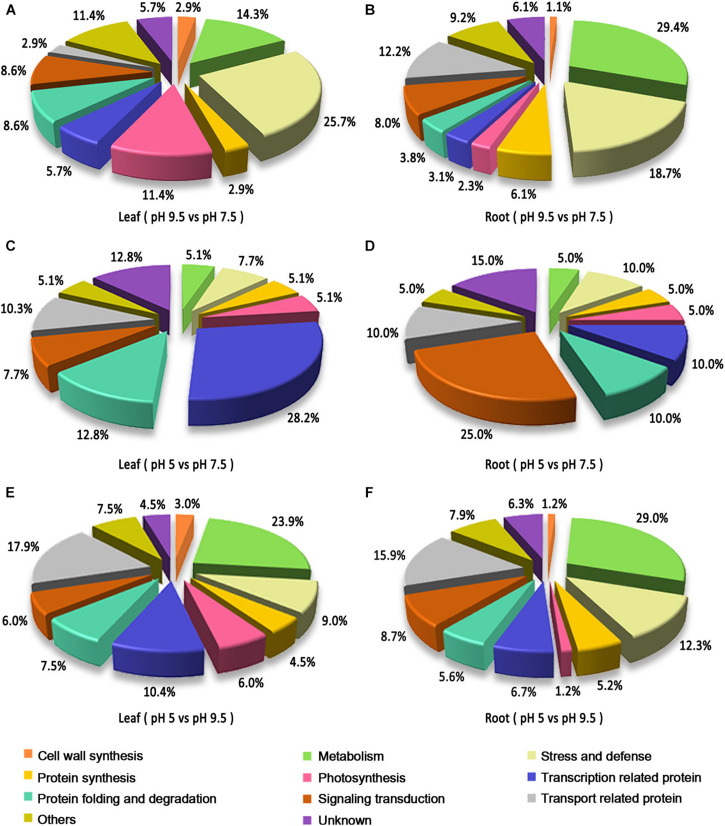
Functional classification of the identified differentially expressed proteins (DEPs) in the leaves and roots of sugar beet in different pH comparison groups. Functional classification of DEPs in leaf **(A)** and root **(B)** in the group of pH 9.5 vs. pH 7.5. Functional classification of DEPs in leaf **(C)** and root **(D)** in the group of pH 5 vs. pH 7.5. Functional classification of DEPs in leaf **(E)** and root **(F)** in the group of pH 5 vs. pH 9.5.

Based on the Gene Ontology (GO), KEGG database, and information from the literature, the functions of the identified DEPs were classified into the categories of metabolism, protein synthesis, transport-related, stress and defense, protein folding and degradation, signaling transduction, cell structure, photosynthesis, transcription-related protein, cell wall synthesis and unknown (Figure 4). In leaves, the prominent functional categories by classifying DEPs in pH 9.5 vs. pH 7.5, pH 5 vs. pH 7.5, and pH 5 vs. pH 9.5 groups were stress and defense, transcription-related, and metabolism, respectively. However, in roots, the most abundant that belonged to the metabolism category in the group’s pH 9.5 vs. pH 7.5 and pH 5 vs. pH 9.5, and the largest proportion of signaling transduction category was found in pH 5 vs. pH 7.5. Moreover, the second highly enriched functional category was significantly different in the groups pH 9.5 vs. pH 7.5 and pH 5 vs. pH 7.5. For example, the next categories in the roots of pH 5 vs. pH 7.5 and pH 9.5 vs. pH 7.5 were transport-related, and stress and defense. The second proportion of pH 9.5 vs. pH 7.5 and pH 5 vs. pH 7.5 in sugar beet leaves was protein folding and degradation, and metabolism. These results indicated that sugar beet responded to different pH environments by regulating the expression of different functional proteins.

To thoroughly analyze the biological pathways of differentially expressed proteins in response to different pH environments, GO term enrichment for DEPs in pH 9.5 vs. pH 7.5, pH 5 vs. pH 7.5, and pH 5 vs. pH 9.5 groups was conducted (Supplementary Figure 3). As expected, the top twenty significantly enriched GO terms in pH 9.5 vs. pH 7.5, pH 5 vs. pH 7.5, and pH 5 vs. pH 9.5 groups exhibited large differences. For the biological process category, in leaves, the most significantly enriched GO terms in pH 9.5 vs. pH 7.5, pH 5 vs. pH 7.5, and pH 5 vs. pH 9.5 groups were negative regulation of molecular function, rRNA modification, and multi-organism process, respectively. In roots, the glucosamine-containing compound catabolic process, cellular response to nitrate, and single-organism localization were the most significantly enriched GO terms for biological process analysis in pH 9.5 vs. pH 7.5, pH 5 vs. pH 7.,5 and pH 5 vs. pH 9.5 groups (Supplementary Figure 3). For the molecular function category, in leaves, the most significantly enriched GO terms in pH 9.5 vs. pH 7.5, pH 5 vs. pH 7.5 and pH 5 vs. pH 9.5 groups were phosphoric diester hydrolase activity, DNA binding, and molecular function regulator, respectively. In roots, chitinase activity was the most significantly enriched GO term at the molecular function level in pH 9.5 vs. pH 7.5, and the most significantly enriched GO term in pH 5 vs. pH 7.5 and pH 5 vs. pH 9.5 was protein heterodimerization activity (Supplementary Figure 3). These data once again prove that different biological pathways are involved in sugar beet response to different soil pH environments.

### Differentially Expressed Proteins Related to Different Sugar Beet Response to the Acidic and Alkaline Environment

**FIGURE 5:**
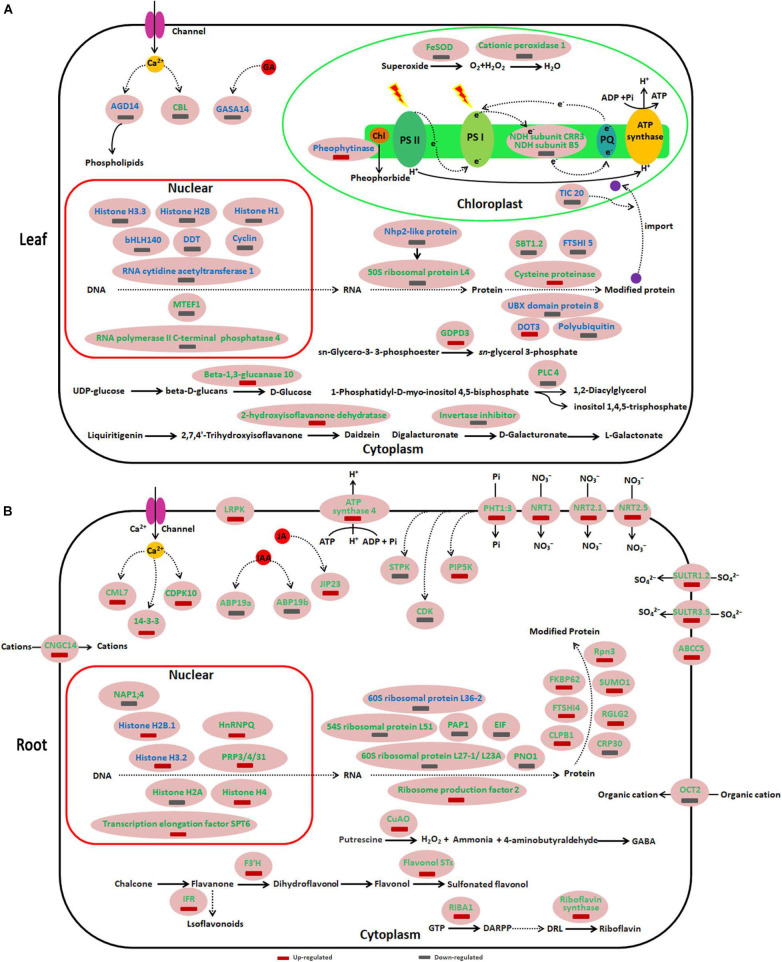
Schematic presentation of key differentially expressed proteins (DEPs) involved in different responses of sugar beet to acid and alkaline pH environment. **(A,B)** represent the DEPs in leaves and roots, respectively. Blue highlighted proteins indicate only increased or decreased in the group of 9.5 vs. pH 7.5. The green highlighted proteins indicate only increased or decreased in the group of 9.5 vs. pH 7.5. ABCC5, ABC transporter C family member 5; ABP19b, Auxin-binding protein19b; AGD14, ADP-ribosylation factor GTPase-activating protein; ARPP, 5′-phosphate; 5-amino-6-ribosylamino-2,4(1H,3H)-pyrimidine DRL, 5′-phosphate; 6,7-dimethyl-8-ribityllumazine; CBL, Calcineurin B-like protein; CDK, cyclin-dependent serine/threonine-protein kinase; CDPK10, Calcium-dependent protein kinase 10; CML7, Calmodulin-7-like; CNGC14, Cyclic nucleotide-gated ion channel 14; CRP30, F-box protein CPR30 isoform X1; CuAO, copper-containing amine oxidases; CLPB1, Chaperone protein ClpB1; DARPP, ARPP, 2,5-diamino-6-ribosylamino-4(3H)-pyrimidinone; DOT3, BTB/POZ domain-containing protein DOT3; EIF, Eukaryotic translation initiation factor-like; F3′H, Flavonoid 3′-hydroxylase; FKBP62, Peptidyl-prolyl cis-trans isomerase FKBP62; FTSHI, Probable inactive ATP-dependent zinc metalloprotease FTSHI; GASA14, Gibberellin-regulated protein 14; GDPDL3, Glycerophosphodiester phosphodiesterase 3; hnRNP Q, Heterogeneous nuclear ribonucleoprotein; IFRL, isoflavone reductase-like protein; JIP23, 23 kDa jasmonate-induced protein; LRPK, Leucine-rich repeat receptor-like serine/threonine-protein kinase; NAP1;4, Nucleosome assembly protein 1;4; Nhp2-like protein, ACA ribonucleoprotein complex subunit 2-like protein; NRT, Nitrate transporter; OCT2, Organic cation/carnitine transporter 2; PAP1, Polyadenylate-binding protein-interacting protein 1; PHT1:3, inorganic phosphate transporter 1-3; PIP5K, Phosphatidylinositol-4-phosphate 5-kinase; PLC4, Phosphoinositide phospholipase C 4; PNO1, Pre-rRNA-processing protein; Prp3, U4/U6 small nuclear ribonucleoprotein; Rpn3, 26S proteasome non-ATPase regulatory subunit 3; SBT1.2, Subtilisin-like protease; SULTR, Sulfate transporter; SUMO1, Small ubiquitin-related modifier 1.

Like the different genotype and growth status of sugar beet under acid (pH 5) and alkaline (pH 9.5) conditions, the DEP_*S*_ of sugar beet in pH 9.5 vs. pH 7.5, pH 5 vs. pH 7.5 were compared in our study. Compared with neutral pH 7.5, the DEP_*S*_ in sugar beet under acidic (pH 5) and alkaline (pH 9.5) conditions were significantly different (Figure 5 and Supplementary Table 5). Several DEP_*S*_ especially identified in pH 9.5 vs. pH7.5 or pH 5 vs. pH 7.5 are listed in Figure 5. In leaves, some DEP_*S*_ belonging to transcription related proteins were especially identified in pH 5 vs. pH 7.5. For example, Histone H3.3, Histone H2B, and Histone H1 were all decreased in the group of pH 5 vs. pH 7.5 but not in that of pH 9.5 vs. pH 7.5 (Figure 5A and Supplementary Table 5). Other proteins related to protein synthesis and protein folding and degradation were also found to be only differently accumulated in pH 9.5 vs. pH 7.5 or pH 5 vs. pH 7.5. 50S ribosomal protein L4 and subtilisin-like protease SBT1.2 only expressed differently in the group of pH 9.5 vs. pH 7.5 in leaves (Figure 5A). Also, two proteins that participated in calcium signaling transduction and GA signaling transduction were downregulated in the group of pH 5 vs. pH 7.5 in leaves (Figure 5A and Supplementary Table 5). Compared with the pH 5 vs. pH 7.5 group, many proteins involved in sugar metabolism, photosynthesis, and other metabolic processes were found to be only differentially expressed in pH 9.5 vs. pH 7.5 (Figure 5A).

In roots, compared with the pH 5 vs. pH 7.5 group, many DEP_*S*_ identified in the pH 9.5 vs. pH 7.5 were especially related to calcium, IAA and JA signaling transduction, transcription related, protein synthesis, and several key metabolic processes (Figure 5B and Supplementary Table 6). Furthermore, it was found that the proteins related to plant nutrient uptake were specifically induced in pH 9.5 vs. pH 7.5, but not changed in pH 5 vs. pH 7.5 (Figure 5B and Supplementary Table 6). For example, phosphate transporter 1-3 and four high-affinity nitrate transporters (NRT1, NRT 2.5, NRT 2.1, and NRT 8.3) were only up-regulated in the roots of pH 9.5 vs. pH 7.5 group.

### Differentially Expressed Proteins Are Related to the Inhibition of Sugar Beet Growth Under Acidic Conditions and the Response of Sugar Beet to Acid Stress

**FIGURE 6:**
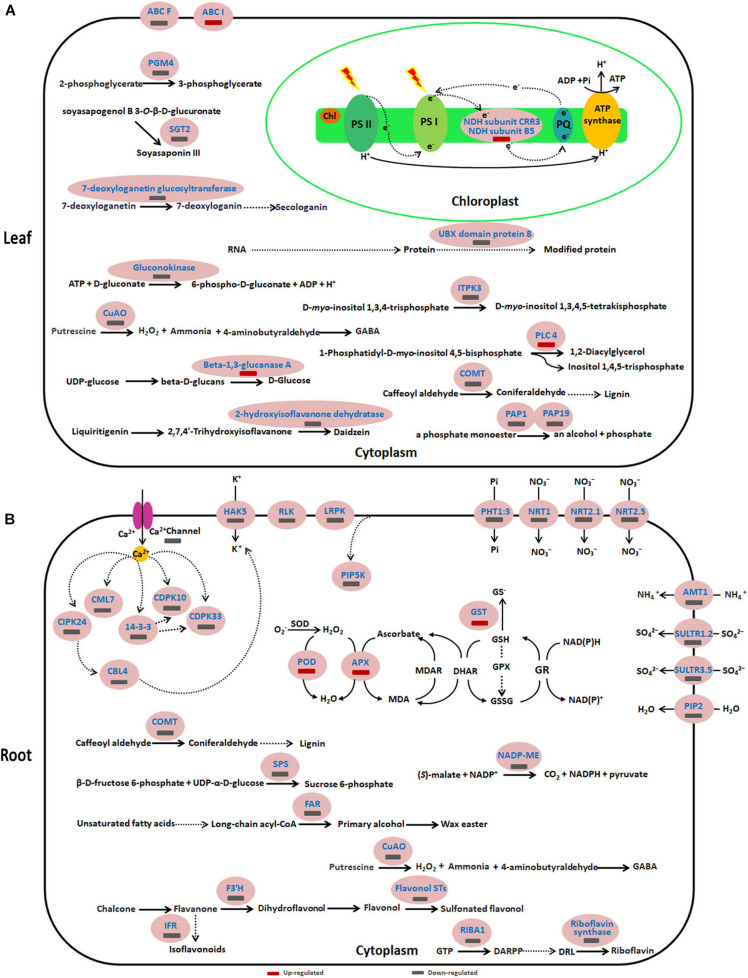
Schematic presentation of key differentially expressed proteins (DEPs) involved in the response of sugar beet to acid stress. **(A,B)** represent the differentially expressed proteins in leaves and roots, respectively. Blue highlighted proteins indicate only increased or decreased in the group of pH 5 vs. pH 9.5. AMT1, Ammonium transporter 1; CBL, Calcineurin B-like protein; CDPK, Calcium-dependent protein kinase; CIPK, CBL-interacting serine/threonine-protein kinase; CML7, Calmodulin-7-like; COMT, caffeic acid 3-O-methyltransferase; CuAO, copper-containing amine oxidases; FAR6, Fatty acyl-CoA reductase 6; FST, Flavonol sulfotransferase; HAK5, High-affinity potassium transporter 5; IFR, isoflavone reductase-like; ITPK3, Inositol-tetrakisphosphate 1-kinase 3; NADP-ME2, NADP-dependent malic enzyme 2; NRT, Nitrate transporter; PAP, purple acid phosphatase; PGM4, phosphoglycerate mutase-like protein 4; PHT1:3, inorganic phosphate transporter 1-3; PIP2-1, Aquaporin PIP2-1; SGT, Soyasapogenol B glucuronide galactosyltransferase; SPS, Sucrose-phosphate synthase; SULTR, Sulfate transporter.

To more comprehensively understand the negative effect of acidic conditions on sugar beet growth and its response mechanism to acid stress, functions, and metabolic pathways analyses were conducted on DEPs in the leaves and roots of pH 5 vs. pH 9.5 group (Figure 6 and Supplementary Tables 7, 8). In leaves, many DEPs participating in several basal biological processes such as lignin synthesis, hydrolysis of phosphate ester, and polyamine metabolism decreased in the condition of pH 5 compared with pH 9.5 (Figure 6A and Supplementary Table 7). For example, copper-containing amine oxidases (CuAO) involved in the polyamine metabolism were inhibited under acidic conditions. These results showed that acidic conditions affected several basic biological processes and led to growth inhibition.

Similar to leaves, the expression of some proteins involved in biological processes such as lignin synthesis, wax easter synthesis, and calcium signaling transduction was inhibited in roots at pH 5 (Figure 6B and Supplementary Table 8). However, key enzymes of the antioxidant enzyme system such as SOD, APX, and GST increased significantly under acidic conditions (Figure 6B and Supplementary Table 8). This indicated that acid stress caused ROS accumulation in sugar beet, and then increased the antioxidant enzyme protein content to respond to oxidative stress caused by excessive accumulation of ROS. Furthermore, it was found that the proteins related to plant nutrient uptake and utilization were significantly decreased in pH 5 compared with pH 9.5 (Figure 6B and Supplementary Table 8). For example, phosphate transporter 1-3 and four high-affinity nitrate transporters (NRT1, NRT 2.5, NRT 2.1, and NRT 8.3) were down-regulated in the pH 5 vs. pH 9.5 group.

### High pH Environment Enhancing the Expression of N Transporters, and Improving N Content in Sugar Beet

**FIGURE 7:**
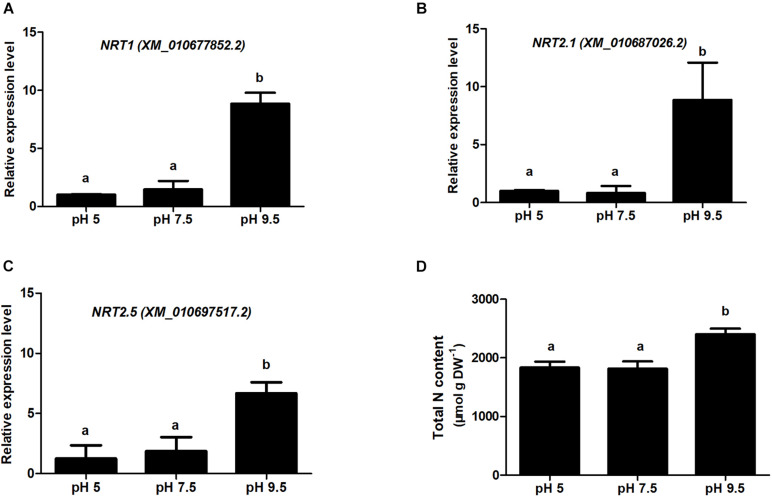
Relative gene expression for *nitrate transporter (NRT)* examined using QRT-PCR and analysis of total nitrogen content in sugar beet under different pH conditions. **(A-C)** indicate the QRT-PCR results of *NRT1*, *NRT2.1*, and *NRT2.5* in the root samples treated with different soil pH. **(D)** indicates the total nitrogen content of sugar beet seedlings under different pH conditions. Different letters indicate significantly different at *p* < 0.05.

Based on the analysis of DEP_*S*_ in the three groups pH 9.5 vs. pH 7.5, pH 5 vs. pH 7.5, and pH 5 vs. pH 9.5 in roots, we found that the protein expression level of sugar beet N transporters was high in alkaline condition (pH 9.5), but low in neutral (pH 7.5), and acid (pH 7.5) condition (Supplementary Tables 6, 8). We thus speculate that alkaline conditions promote the absorption of N nutrients and enhance sugar beet growth. Subsequently, we used QRT-PCR and semi-quantitative RT-PCR to verify the expression of three N transporters (NTR1, NRT2.1, and NRT2.5) at the transcription level in roots (Figures 7A-C and Supplementary Figure 4). Consistent with the proteomics results, the expression levels of these genes were relatively high under alkaline conditions compared with pH 5 or pH 7.5 (Figures 7A-C and Supplementary Tables 6, 8). Furthermore, the contents of total N in sugar beet under different pH conditions were analyzed (Figure 7D). The highest N levels in sugar beet were detected at pH 9.5, which was significantly higher than pH5 and pH 7.5. These results showed that high pH conditions enhanced sugar beet growth by regulating the expression of N transporters and N absorption.

## Discussion

Soil pH is one of the important environmental factors that affect plant growth and root development. Therefore, understanding the physiological and molecular mechanisms of plant response to different pH environments can help to increase plant resistance to non-optimal pH conditions through genetic improvement. Generally, the optimum pH for different kinds of plants is quite different, and unsuitable pH will inhibit the growing. For example, the optimal pH level for flax growth is about 5.0-5.5, and unfavorably low pH results in crucial damage to plants (Dmitriev et al., 2019). This study shows that sugar beet has the best growth state in high pH environment, and low soil pH affects sugar beet photosynthesis. Similarly, phenotypic and physiological analyses revealed that the optimum pH of iron walnut was 4-5 (Luo and Liu, 2019), and the net photosynthetic rate significantly declined under non-optimal pH conditions. Moreover, it has recently been reported that low pH affected ROS and MG metabolisms in *Citrus* roots and leaves, and the negative effect was more significant in roots than in leaves (Long et al., 2019). However, low pH-treated sugar beet enhanced the activities of APX and SOD for removing ROS. These studies indicated that activities of these enzymes related to ROS metabolisms may play a key role in low pH response of sugar beet. Moreover, changes of protein abundance play a vital role in plant responses to different pH environments. Our research revealed that the difference mechanisms to response different pH in the leaves and roots of sugar beet might be attributed to the differential accumulation of some responsive proteins. Thus, we discussed the DEPs in roots and leaves in sugar beet response to different pH conditions, respectively.

### DEPs Related to Sugar Beet Different Response to Acid and Alkalinity in Leaves

By comparing the DEPs between pH 9.5 vs. pH 7.5 and pH 5 vs. pH 7.5, it was found that the changes of several proteins were significantly different in the leaves of the two groups. Usually, plants maintain sophisticated gene transcription programs to regulate their development and response to the environment. In this study, three histone proteins (histone H3.3, H2B, and H1) involved in transcriptional regulation were especially decreased in the leaves of pH 5 vs. pH 7.5. Histone organizes DNA into nucleosomes and thus modulates DNA exposure during transcriptional regulation. Recently, H3.3, a major variant of histone H3, has been implicated in modulating abiotic stresses (cold, heat, NaCl) response in *Gossypium hirsutum* (Qanmber et al., 2019). Our studies indicated that three histone proteins especially changed in pH 5 vs. pH 7.5 might be involved in the specific response changes induced by low pH in leaves.

The ADP-RIBOSYLATION FACTOR GTPase-ACTIVATING PROTEIN (AGD), is one of the key regulators of vesicle transport and is a diverse family of proteins. It has also been reported that AGD can participate in calcium signaling through binding phospholipids in a calcium-dependent manner (Dümmer et al., 2016). Therefore, the decreasing AGD14 in pH 5 vs. pH 7.5 may indicate that low pH declined AGD14-mediated phospholipids binding depends on Ca^2+^, and the increase of free phospholipids play an important signaling role in the response of plants to low pH stress (Heilmann and Heilmann, 2015). Phospholipase C (PLC) predominantly hydrolyzes phosphatidylinositol-4,5-biphosphates into the second messenger’s diacylglycerol (DAG) and inositol 1,4,5-trisphosphate. OsPLC1 was found to hydrolyze phosphatidylinositol-4-phosphate (PtdIns4P) and elicited stress-induced Ca^2+^ signals regulating salt tolerance (Li et al., 2017). Thus, the decrease of PLC 4 in the leaves of pH 9.5 vs. pH 7.5 indicated that the optimal pH condition tended to close the PLC-mediated stress signaling pathway.

β-1,3-glucanase hydrolyses β-1,3-glucans are the main components of the cell wall. By degrading callose, β-1,3-glucanases are also involved in various physiological and developmental processes, such as cell elongation, cell division, and pollen germination (Wojtasik et al., 2013). It has been shown that *SbGlu1*, encoding a β-1,3-glucanase for callose degradation, played important roles in sorghum Al resistance in acid soils through the modulation of callose deposition (Gao et al., 2019). β-1,3-glucanases 10 in leaves was significant up-regulated in pH 9.5 vs. pH 7.5 in our study, demonstrating that a high alkaline environment promotes sugar beet seedling growth by increasing lignin deposition.

Glycerophosphodiester phosphodiesterases (GDPDs) are enzymes involved in the degradation of glycerophosphodiesters into *sn*-glycerol-3-phosphate. Glycerol-3-phosphate can also be dephosphorylated to release glycerol, thus serving as a Pi source for Pi starved cells (Mehra and Giri, 2016). *OsGDPDs2* have been reported to be induced under Pi deficiency and impart significant changes in glycerolipids and fatty acids levels in rice (Mehra et al., 2019). Furthermore, overexpression of *OsGDPDs2* led to increased GDPD activity, Pi content, and biomass in rice under Pi starvation. GDPD3 also has an especially increasing abundance in the leaves of pH 9.5 vs. pH 7.5. Thus, GDPD3 might participating in increasing biomass under pH 9.5 conditions through remobilization of Pi from phospholipids.

### DEPs Related to Sugar Beet Different Response to Acid and Alkalinity in Roots

Differentially expressed proteins changed significantly between the groups of pH 9.5 vs. pH 7.5 and pH 5 vs. pH 7.5 in the roots. Polyamines (PAs) are essential metabolites in plants, involved in a wide range of crucial cellular processes. Copper-containing amine oxidases (CuAOs) catalyze the catabolism of PAs (Alharbi et al., 2020). The action of CuAOs on di-amine precursor putrescine (Put) yields 4-aminobutyraldehyde, which then cyclizes to gamma-aminobutyric acid (GABA). It has also been reported that GABA modulation of aluminum-activated malate transporter (ALMT) activity resulted in altered root growth and tolerance to alkaline pH, acid pH, and aluminum ions (Ramesh et al., 2015). Also, GABA can enter the Krebs cycle and promote plant growth (Alharbi et al., 2020). Therefore, compared with the pH 5 vs. pH 7.5 group, the abundance of two CuAOs increased only in roots under pH 9.5 vs. pH 7.5 conditions, which may lead to a higher accumulation of GABA and more energy supply to stimulate the growth of sugar beet.

Riboflavin serves as a precursor for flavocoenzymes (FMN and FAD) and is required for photosynthesis, mitochondrial electron transport, fatty acid oxidation, and biosynthesis of numerous secondary metabolites. The processes of riboflavin biosynthesis are involved in several reactions, and bifunctional riboflavin biosynthesis protein RIBA1 and riboflavin synthase are two key enzymes that synthesize riboflavin (Hiltunen et al., 2012). Bifunctional riboflavin biosynthesis protein RIBA1 and riboflavin synthase are all only up regulated in the group of pH 9.5 vs. pH 7.5 in roots. It is demonstrated that alkaline pH condition increased riboflavin synthesis, which might be involved in the adaptability of sugar beet to a high pH environment.

Flavonoids, a class of important secondary metabolites, are classified into different subgroups, such as flavones, flavonols, flavanones, and proanthocyanidins (Deng et al., 2018). Flavonoids play significant roles in plant antioxidant activity, UV-light protection, and defense against phytopathogens. We found that three key enzymes involved in flavonoid biosynthesis and metabolism were shown to increase only in the group of pH 9.5 vs. pH 7.5. The widespread presence of flavonoids at the cellular level is crucial for the plant’s response to environmental cues. For example, UV light induces the synthesis of flavonoids with higher hydroxylation levels, which play an important role in ROS-detoxification in plant cells (Deng et al., 2018). Thus, the high level of isoflavone reductase-like, flavonol sulfotransferase-like, and flavonoid 3′-hydroxylase in pH 9.5 will increase the content of isoflavonoids, flavonols, and sulfonated flavonols. These accumulated substances could promote the growth of sugar beet and enhance its adaptability to a high pH environment.

Phosphorus (P) and nitrogen (N) are essential macronutrients for plant growth and development. The primary source of P and N took up by plants is inorganic phosphate (Pi) and nitrate (NO^3–^) in agricultural soils. Specific transport systems are essential for taking up Pi and NO^3–^, and the transport of Pi and NO^3–^ are mediated largely by Pi transporters and NO^3–^ transporters. PHT1 and NRT transporter families play key roles in P and N acquisition, respectively, and them within plants (Plett et al., 2010; Chen et al., 2011). Several transporters (NRT1, NRT2.1, NRT2.5, and PHT1;3) involved in these processes have only been upregulated in the roots of pH 9.5 vs. pH 7.5. These findings suggest Pi and N transporters belonging to the PHT1 and NRT families in sugar beet roots may be involved in high pH promoting growth through enhancing the absorption of Pi and N.

### DEPs Related to Related to the Inhibition of Sugar Beet Growth Under Acidic Conditions and the Response of Sugar Beet to Acid Stress in Leaves

The functions of the DEP_*S*_ in the pH5 vs. pH9.5 group and their related physiological and metabolic processes were also systematically discussed. Isoflavonoids act as signaling molecules for regulating plant growth and development. Isoflavones are early products of the isoflavonoid biosynthesis pathway, and 7-deoxyloganetin glucosyltransferase is essential for isoflavone daidzein biosynthesis (Akashi et al., 2005). Therefore, the reduced abundance of 7-deoxyloganetin glucosyltransferase in pH 5 demonstrated that an acidic environment might affect the growth of sugar beet by reducing the synthesis of isoflavonoids.

Purple acid phosphatase (PAP) catalyzes the hydrolysis of Pi from various phosphate monoesters and anhydrides in the acidic pH range. The function of PAP in plants was involved in recycling Pi from expendable intracellular organophosphate pools or mobilizing Pi from the external organophosphates in soil (Robinson et al., 2012). For example, AtPAP26 is a principal contributor to Pi stress-inducible ATPase activity and plays an important role in the *Arabidopsis* Pi metabolism (Robinson et al., 2012). Compared with pH 9.5, the reducing level of PAP1 and PAP 29 in leaves of pH 5 caused by low pH may inhibit the Pi recycling in the leaves of sugar beet and affect plant growth.

Lignin is the major structural component of secondarily thickened plant cell walls that is involved in the mechanical strength of stems and trunks. In the lignin biosynthesis process, caffeic acid *O*-methyltransferase (COMT) is a bifunctional enzyme responsible for converting caffeic acid to ferulic acid and 5-hydroxyferulic acid to sinapic acid (Guo et al., 2001). It has been reported that down-regulation of barley caffeic acid O-methyltransferase reduced stem lignin content and dramatically changed lignin structure. Interestingly, it has been found that it is responsible for the production of melatonin. Melatonin (*N*-acetyl-5-methoxytryptamine) has been characterized as an important bioactive molecule that is implicated in plant growth and stress response (Hardeland, 2015). In *Arabidopsis*, melatonin could induce salinity or cold stress-responsive genes. It has been shown to support abiotic stress resistance and to delay leaf senescence. Therefore, the decreased COMT protein in the leaves of pH 5 vs. pH 9.5 not only affected the synthesis of lignin but also the response of plants to stress by reducing the melatonin content.

### DEPs Related to Related to the Inhibition of Sugar Beet Growth Under Acidic Conditions and the Response of Sugar Beet to Acid Stress in Roots

Calcium is one of the essential nutrients for the growth and development of plants, and it plays a key role in plants under different developmental cues and various stress conditions as a major secondary-messenger molecule. External stimuli trigger specifically intracellular spatial and temporal [Ca^2+^] cyt variations in plant cells. Calcium sensors perceive [Ca^2+^] cyt variations and transmit resulting signals to the downstream effectors to activate specific stress responses. The Ca^2+^ sensors involved in plant stress signaling pathways are represented by the calcium-dependent protein kinases (CDPKs), calmodulins (CaMs), calmodulin-like proteins (CMLs), and calcineurin B-like proteins (CBLs) and their interacting kinases (CIPKs). For example, AtCML9 plays an essential role in modulating responses to salt stress through its effects on the ABA-mediated pathways (Magnan et al., 2008). CDPKs are involved in supporting plant adaptation under drought, salinity, and heat and cold stress environments. *AtCDPK27* and *AtCDPK12* expression were all induced by NaCl, and mutants of CPK27 or *CPK12*-RNAi plants were much more sensitive to salt stress than the wild-type plant (Magnan et al., 2008). Compared with pH 9.5, pH 5 significantly reduced the abundance of a series of Ca^2+^ sensors in sugar beet roots (CDPK10, CDPK23, CML7, CIPK24, and CBL4). These studies proved that the calcium signaling pathway was significantly inhibited under acidic conditions. Furthermore, the calcium-dependent activity of this CDPK was shown to be stimulated by 14-3-3 proteins, which are phosphopeptide-binding proteins (van Kleeff et al., 2018). Interestingly, the level of 14-3-3 protein decreased significantly at pH 5. This phenomenon demonstrated that low pH not only affected calcium sensors but also other regulatory proteins participating in calcium signal transduction.

Sucrose phosphate synthase (SPS) has been proved to play an important role in carbon metabolism. It is the rate-limiting enzyme in sucrose synthesis and affects sucrose accumulation in plants (Wang et al., 2017b). It is reported that overexpression of a maize *SPS* gene in potatoes enhanced the rate of photosynthesis and increased yield (Ishimaru et al., 2008). Therefore, the low level of SPS protein in pH 5 may inhibit plant growth by affecting carbon metabolism. Furthermore, three nitrate transporters were significantly decreased in the roots of the pH 5 vs. pH 9.5 group. In consistent, the N content of sugar beet in pH 5 was significantly lower than that in pH 9.5, indicating low pH affected N absorption by inhibiting nitrate transporters.

## Conclusion

In summary, this study found that sugar beet can adapted to high pH, and the acid condition seriously inhibited its growth. Low soil pH can exert strongly negative effects on photosynthesis and a high pH promotes it in sugar beet seedlings. Moreover, sugar beet enhanced antioxidant activities and synthesized higher levels of soluble organic solutes to cope with acid stress. Furthermore, based on TMT quantitative proteomic analysis, we identified proteins with abundance alterations in response to different pH environment. Several key proteins and pathways related to the sugar beet response to different pH conditions were identified. It was found that the growth of sugar beet could be promoted by increasing the abundance of NRT transporter and enhancing nitrogen absorption under high pH. Our data thus prove that sugar beet has different mechanisms to respond different pH environment. Future studies focusing on characterizing the biological significance of these key proteins will be highly valuable in designing molecular breeding or engineering programs for enhancing sugar beet tolerance to non-optimal pH.

## Data Availability Statement

The datasets presented in this study can be found in online repositories. The names of the repository/repositories and accession number(s) can be found below: http://www.proteomexchange.org/, PXD024589.

## Author Contributions

GG and YW designed the experiments. GG, YW, GW, CL, QW, and LY conducted most of the physiological and biochemical analyses. YW, GG, and GW performed the proteome analyses. YW, GG, and PS contributed to writing and revised the manuscript. All authors read and approved the final manuscript.

## Conflict of Interest

The authors declare that the research was conducted in the absence of any commercial or financial relationships that could be construed as a potential conflict of interest.

## Footnotes

1 https://github.com/tanghaibao/GOatools

## Supplementary Material

Supplementary Figure 1 Principal components analysis (PCA) on proteomic data of sugar beet treated with three pH environments.

Supplementary Figure 2 Cluster analysis of differentially expressed proteins (DEPs) in the leaves and roots of sugar beet in different pH comparison groups.

Supplementary Figure 3 The GO (Gene Ontology) enrichment analysis of identified differentially expressed proteins (DEPs) in the leaves and roots of sugar beet in different pH comparison groups.

Supplementary Figure 4 Relative gene expression for *nitrate transporter (NRT)* examined using semi-quantitative RT-PCR analysis in sugar beet under different pH conditions.

Supplementary Table 1 List of the QRT-PCR primer.

Supplementary Table 2 Peptide information identified by mass spectrometry in leaves.

Supplementary Table 3 Peptide information identified by mass spectrometry in roots.

Supplementary Table 4 Protein information identified by mass spectrometry in leaves and roots.

Supplementary Table 5 Identification of differential expression proteins (DEPs) in the leaves of pH 9.5 vs. pH 7.5 and pH 5 vs. pH 7.5 groups.

Supplementary Table 6 Identification of differential expression proteins (DEPs) in the roots of pH 9.5 vs. pH 7.5 and pH 5 vs. pH 7.5 groups.

Supplementary Table 7 Identification of differential expression proteins (DEPs) in the leaves of pH 5 vs. pH 9.5 group.

Supplementary Table 8 Identification of differential expression proteins (DEPs) in the roots of pH 5 vs. pH 9.5 group.
