# Supplementary material for: Physiological and Proteomic Analysis of Different Molecular Mechanisms of Sugar Beet Response to Acidic and Alkaline pH Environment
Source: Front Plant Sci. 2021 Jun 9;12:682799. doi: 10.3389/fpls.2021.682799 (PMC8220161; doi:10.3389/fpls.2021.682799)
Supplement: Supplementary Figure 1 — Principal components analysis (PCA) on proteomic data of sugar beet treated with three pH environments. [file Data_Sheet_1.DOCX]

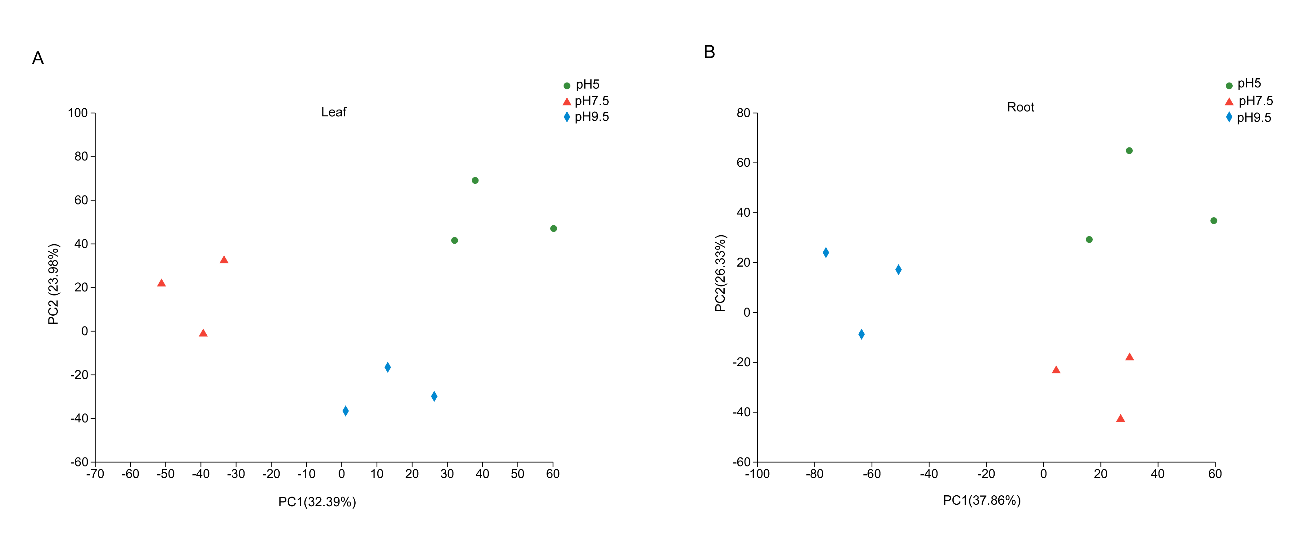


**Figure S1****.** Principal components analysis (PCA) on proteomic data of sugar beet treated with three pH environments. (A) and (B) indicated the PCA analysis of sugar beet leaves or roots under different pH treatments, respectively.
