# Supplementary material for: Physiological and Proteomic Analysis of Different Molecular Mechanisms of Sugar Beet Response to Acidic and Alkaline pH Environment
Source: Front Plant Sci. 2021 Jun 9;12:682799. doi: 10.3389/fpls.2021.682799 (PMC8220161; doi:10.3389/fpls.2021.682799)
Supplement: Supplementary Figure 2 — Cluster analysis of differentially expressed proteins (DEPs) in the leaves and roots of sugar beet in different pH comparison groups. [file Data_Sheet_2.DOCX]

**Figure S2**. Cluster analysis of differentially expressed proteins (DEPs) in the leaves and roots of sugar beet in different pH comparison groups. Cluster analysis of DEPs in root (A) and leaf (B) in the group of pH 9.5 *vs* pH 7.5. Cluster analysis of DEPs in root (E) and leaf (F) in the group of pH 5 *vs* pH 9.5. Cluster analysis of DEPs in root (C) and leaf (D) in the group of pH 5 *vs* pH 7.5.
