# Supplementary material for: Physiological and Proteomic Analysis of Different Molecular Mechanisms of Sugar Beet Response to Acidic and Alkaline pH Environment
Source: Front Plant Sci. 2021 Jun 9;12:682799. doi: 10.3389/fpls.2021.682799 (PMC8220161; doi:10.3389/fpls.2021.682799)
Supplement: Supplementary Figure 3 — The GO (Gene Ontology) enrichment analysis of identified differentially expressed proteins (DEPs) in the leaves and roots of sugar beet in different pH comparison groups. [file Data_Sheet_3.DOCX]

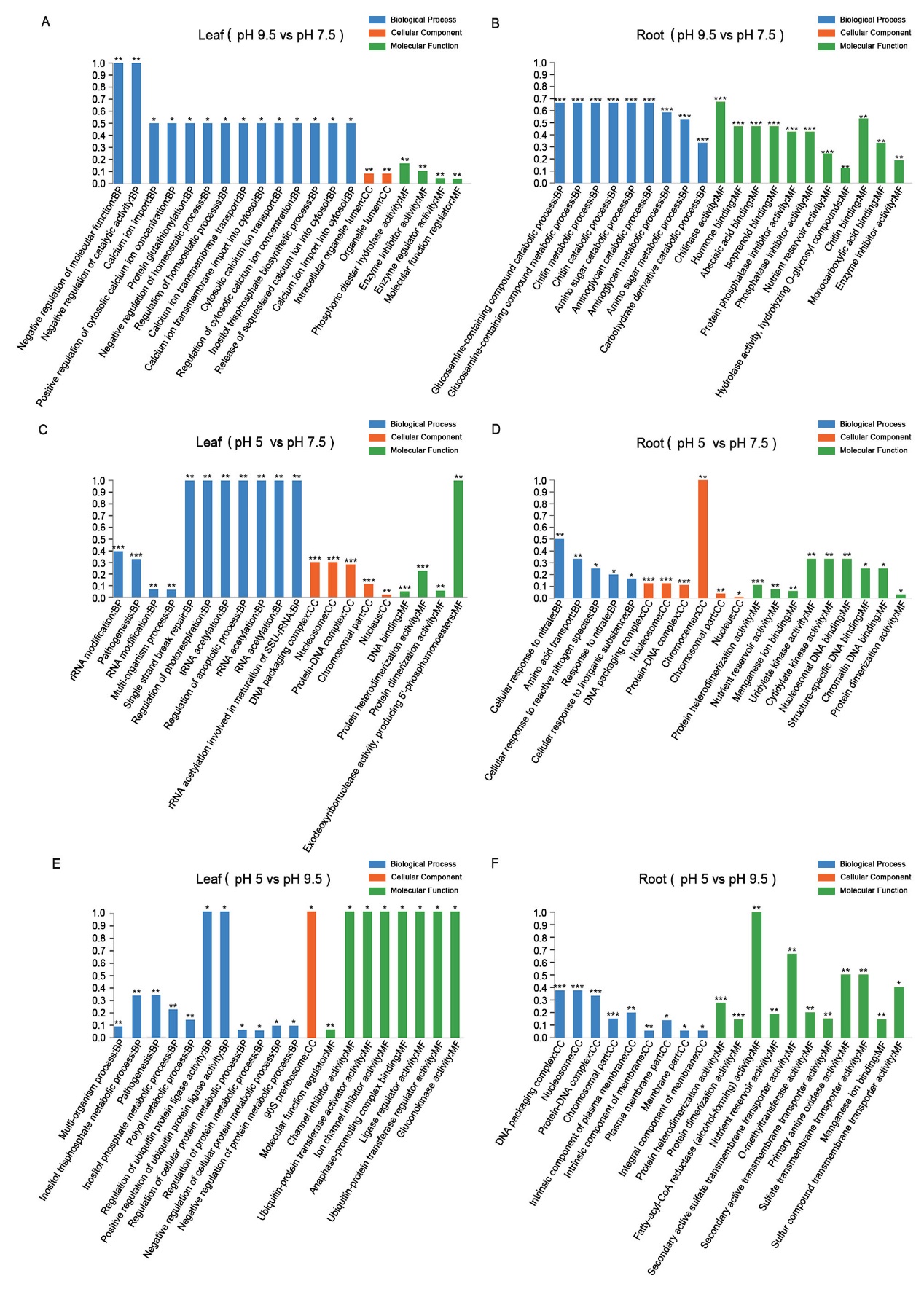


**Figure S3**. The GO (Gene Ontology) enrichment analysis of identified differentially expressed proteins (DEPs) in the leaves and roots of sugar beet in different pH comparison groups. GO enrichment analysis of DEPs in leaf (A) and root (B) in the group of pH 9.5 *vs* pH 7.5. GO enrichment analysis of DEPs in leaf (C) and root (D) in the group of pH 5 *vs* pH 7.5. GO enrichment analysis of DEPs in leaf (E) and root (F) in the group of pH 5 *vs* pH 9.5.
