# Supplementary material for: Physiological and Proteomic Analysis of Different Molecular Mechanisms of Sugar Beet Response to Acidic and Alkaline pH Environment
Source: Front Plant Sci. 2021 Jun 9;12:682799. doi: 10.3389/fpls.2021.682799 (PMC8220161; doi:10.3389/fpls.2021.682799)
Supplement: Supplementary Figure 4 — Relative gene expression for nitrate transporter (NRT) examined using semi-quantitative RT-PCR analysis in sugar beet under different pH conditions. [file Data_Sheet_4.DOCX]

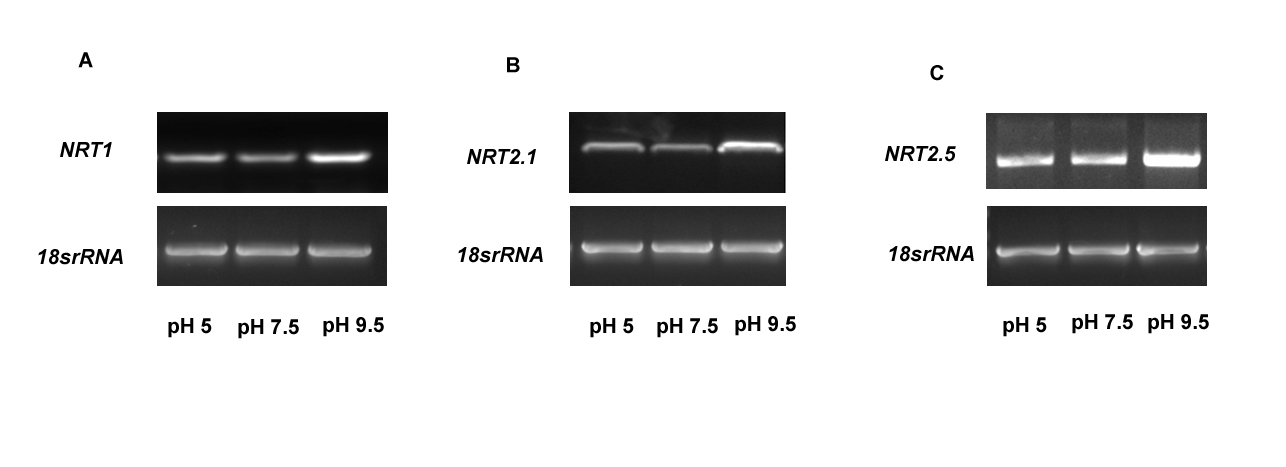


**Figure S4.** Relative gene expression for *nitrate transporter (NRT)* examined using semi-quantitative RT-PCR analysis in sugar beet under different pH conditions. (A, B and C) indicate the semi-quantitative RT-PCR results of *NRT1*, *NRT2.1*, and *NRT2.5* in the root samples treated with different soil pH.
