# Supplementary material for: Physiological and Proteomic Analysis of Different Molecular Mechanisms of Sugar Beet Response to Acidic and Alkaline pH Environment
Source: Front Plant Sci. 2021 Jun 9;12:682799. doi: 10.3389/fpls.2021.682799 (PMC8220161; doi:10.3389/fpls.2021.682799)
Supplement: Supplementary Table 1 — List of the QRT-PCR primer. [file Table_1.DOCX]

**Table S1.** List of the QRT-PCR primer

| **Primer name** | **Primer sequence** |
| --- | --- |
| NRT2.5-F | 5- CTTCCAGTGGATGCAGAGCA-3 |
| NRT2.5-R | 5- CCAACTAAGTCACAGGCGGT-3 |
| NRT2.1-F | 5-GAAACGTGGGAGCAGCATTG-3 |
| NRT2.1-R | 5-TGTTGGTGTACTCGGACGTG-3 |
| NRT1-F | 5-AGTTTGGCTCGTGTACTCCG-3 |
| NRT1-R | 5-CACCAAAACTGTCACGGCTG-3 |
| 18S rRNA-F | 5-CCCCAATGGATCCTCGTTA-3 |
| 18S rRNA-R | 5-TGACGGAGAATTAGGGTTCG-3 |
